# *SYMMETRIC PETALS 1* Encodes an ALOG Domain Protein that Controls Floral Organ Internal Asymmetry in Pea (*Pisum sativum* L.)

**DOI:** 10.3390/ijms21114060

**Published:** 2020-06-05

**Authors:** Liang He, Yawen Lei, Xin Li, Qincheng Peng, Wei Liu, Keyuan Jiao, Shihao Su, Zhubing Hu, Zhenguo Shen, Da Luo

**Affiliations:** 1 State Key Laboratory of Biocontrol and Guangdong Key Laboratory of Plant Resources, School of Life Sciences, Sun Yat-sen University, Guangzhou 510275, China; heliang6@mail2.sysu.edu.cn (L.H.); leiyw3@mail.sysu.edu.cn (Y.L.); pengqch@mail2.sysu.edu.cn (Q.P.); liuwei61611@gmail.com (W.L.); jiaoky@mail2.sysu.edu.cn (K.J.); sushihao@mail2.sysu.edu.cn (S.S.); dluo@sibs.ac.cn (D.L.); 2 College of Life Sciences, Laboratory Center of Life Sciences, Nanjing Agricultural University, Nanjing 210095, China; zgshen@njau.edu.cn; 3 Institute of Plant Stress Biology, State Key Laboratory of Cotton Biology, Department of Biology, Henan University, Kaifeng 475004, China; zhubinghu@henu.edu.cn

**Keywords:** ALOG family, *COCHLEATA*, IN asymmetry, LSH3, *SYMMETRIC PETALS 1*, *Pisum sativum*

## Abstract

In contrast to typical radially symmetrical flowers, zygomorphic flowers, such as those produced by pea (*Pisum sativum* L.), have bilateral symmetry, manifesting dorsoventral (DV) and organ internal (IN) asymmetry. However, the molecular mechanism controlling IN asymmetry remains largely unclear. Here, we used a comparative mapping approach to clone *SYMMETRIC PETALS 1* (*SYP1*), which encodes a key regulator of floral organ internal asymmetry. Phylogenetic analysis showed that SYP1 is an ortholog of *Arabidopsis thaliana* LIGHT-DEPENDENT SHORT HYPOCOTYL 3 (LSH3), an ALOG (Arabidopsis LSH1 and Oryza G1) family transcription factor. Genetic analysis and physical interaction assays showed that COCHLEATA (COCH, *Arabidopsis* BLADE-ON-PETIOLE ortholog), a known regulator of compound leaf and nodule identity in pea, is involved in organ internal asymmetry and interacts with SYP1. *COCH* and *SYP1* had similar expression patterns and COCH and SYP1 target to the nucleus. Furthermore, our results suggested that COCH represses the 26S proteasome-mediated degradation of SYP1 and regulates its abundance. Our study suggested that the COCH-SYP1 module plays a pivotal role in floral organ internal asymmetry development in legumes.

## 1. Introduction

The precise regulation of cellular processes during organ development is important in plants and gives rise to the enormous diversity of plant forms. After the initiation of organogenesis, organ identities are determined and distinct organs with characteristic shapes and sizes develop [1]. Most of the legume plants in the Papilionoideae family have zygomorphic flowers (one plane of floral symmetry), providing an excellent experimental system for investigating the molecular mechanisms of organogenesis.

In Papilionoideae legumes, the zygomorphic flowers possess a prominent corolla with three petal types and their floral organs have dorsoventral (DV) and internal (IN) asymmetry [2,3]. During flower development, petal primordia are uniform in size through the middle stage but take on different shapes and symmetries in late stages [2]. The dorsal petal enlarges and remains bilaterally symmetrical, but the lateral and ventral petals remain small and become asymmetrical [2,3].

Floral zygomorphy is a complex trait and genetic analyses have shown that different factors control the DV and IN asymmetry in legumes [3,4,5,6]. *CYCLOIDEA*-like TCP family genes confer the dorsal and lateral identities in *Lotus japonicus* and pea (*Pisum sativum* L.) [3,4]. Several loci in pea, such as *BIG ORGANS* (*BIO*), *ELEPHANT LEAVES* (*ELE1*), and *SYMMETRIC PETALS 1* (*SYP1*), establish IN asymmetry of the lateral and ventral petals [3,5,6]. In the *syp1* mutant flowers, the lateral and ventral petals have abnormal bilaterally symmetric shapes [3]. However, the molecular identity and mechanism of action of *SYP1* in controlling IN asymmetry have not been investigated.

The ALOG family transcription factors, named after the Arabidopsis LIGHT-DEPENDENT SHORT HYPOCOTYL 1 (LSH1) and rice (*Oryza sativa*) G1 proteins, are widely distributed in plants, share a conserved ALOG domain, and are key regulators of plant organ development [7,8,9,10,11,12,13,14,15]. However, functional studies of ALOG family genes have only been conducted in a few species, including rice, Arabidopsis, and tomato (*Solanum lycopersicum*). *TAWAWA1* (*TAW1*) and *G1* are two rice ALOG genes; TAW1 regulates inflorescence development and G1 represses the growth of the sterile lemma in the spikelet [8,10]. In addition, another ALOG gene *TRIANGULAR HULL1* (TH1) affects spikelet morphogenesis, grain shape, and yield in rice [12]. In Arabidopsis, *LSH3* and *LSH4* encode ALOG domain proteins that are involved in organ boundary formation [16]. In tomato, the ALOG protein TERMINATING FLOWER (TMF) physically interacts with BLADE-ON-PETIOLE (BOP) orthologs to regulate flower and leaf development [11,17,18]. A recent study reported that *TfALOG3*, a member of the ALOG family, controls the development of corolla neck, which may serve as mechanical protection from nectar robbers in *Torenia fournieri,* and *LjALOG1* positively regulates nodulation in *L. japonicus* [15,19]. However, the functions of the ALOG family genes remain largely unknown, especially in legumes.

In this study, we cloned *SYP1* via a comparative genomics approach and found that SYP1 encodes an ALOG protein. Genetic analysis and physical interaction assays showed that COCHLEATA (COCH, *Arabidopsis* BLADE-ON-PETIOLE ortholog), a master regulator of compound leaf and flower development and nodule organogenesis [20,21], interacts with SYP1 to regulate floral organ internal asymmetry in pea. Furthermore, we showed that COCH promotes SYP1 protein stability. Taken together, our results identified a COCH-SYP1 module that functions in the control of floral organ internal asymmetry and nodule development in pea.

## 2. Results

### 2.1. Phenotypes of Coch and syp1 Mutants with Defects in Organ Internal Asymmetry

**Figure 1:**
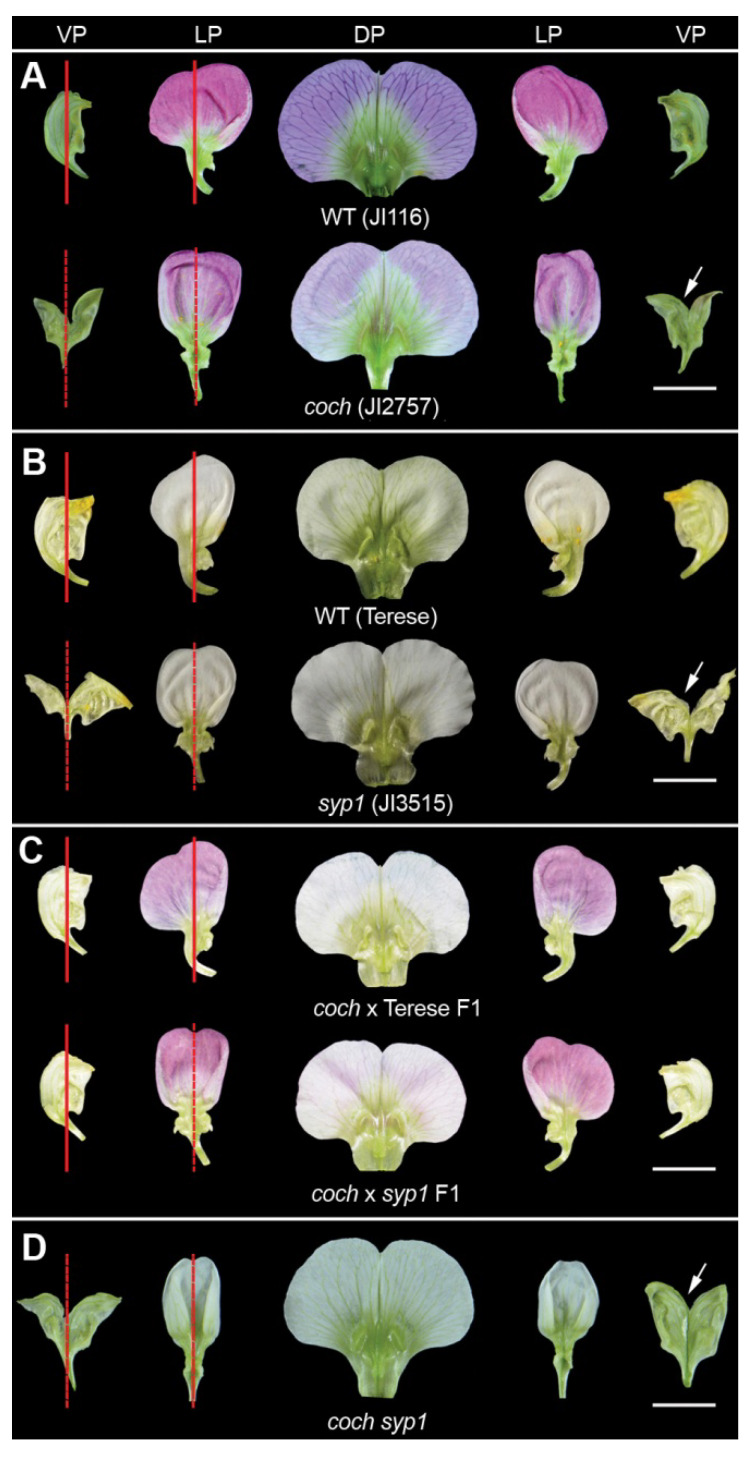
Phenotypes of the *cochleata* (*coch*) and *symmetric petals* (*syp1)* mutants in pea. (**A**) Petals of the wild type (JI116) and the *coch* mutant (JI2757) possess dorsal-ventral (DV) differentiation. (**B**) Petals of the wild type (Terese) and the *syp1* mutant (JI2757) possess DV differentiation. (**C**) Petals of the F_1_ plants (*coch* × Terese, *coch* × *syp1*) possess DV differentiation. (**D**) Petals of the *coch syp1* double mutant possess DV differentiation. The red lines indicate the internal (IN) asymmetry and the dotted lines indicate the abolishment of IN asymmetry. The arrows indicate the cutting at the ventral petals so as to flatten the petals. DP, the dorsal petal; LP, the lateral petal; VP, the ventral petal. (**A**–**D**) Scale bar = 1 cm.

To investigate the genetic control of IN asymmetry and identify the factors interacting with *SYP1*, different pea mutants were screened and analyzed to identify the loci that control floral organ shape [3,22]. We identified a well characterized mutant with defective IN asymmetry, named *coch* for the wild-type gene (*COCH*), encoding ortholog of *Arabidopsis* BLADE-ON-PETIOLE (BOP), which has been well characterized for its role in compound leaf and flower development and nodule organogenesis [20,21]. Interestingly, flowers in the *coch* mutants displayed symmetrical lateral and ventral petals similar to the *syp1* mutant (Figure 1A,B). From the figures of the reports, we found that other lines of *coch* mutants also displayed symmetrical lateral and ventral petals [20,21], indicating that *COCH* plays a pivotal role in organ internal asymmetry.

In Virus-Induced Gene Silencing (VIGS)-*COCH* silenced plants [23], 15.46% and 9.28% of flowers displayed partial or complete defects of organ internal asymmetry, respectively (Supplementary Figure S1). As in the *coch* mutant in pea, the mutants of the *COCH* ortholog in *L. japonicus* show similar abnormal flowers with symmetric lateral and ventral petals [24].

To test whether *COCH* and *SYP1* interact in the control of IN asymmetry, we crossed the *coch* mutant with the *syp1* mutant. The F_1_ plants (*coch/+ syp1/+*) showed a weak phenotype with symmetrical lateral petals and normal ventral petals (Figure 1C), indicating that the two genes may interact in the control of IN asymmetry of lateral petals. In the F_2_, the *coch syp1* double mutants displayed phenotypes similar to that of the *syp1* and *coch* mutants, in that all the petals became symmetrical (Figure 1D).

### 2.2. Molecular Cloning of SYP1 in Pea

**Figure 2:**
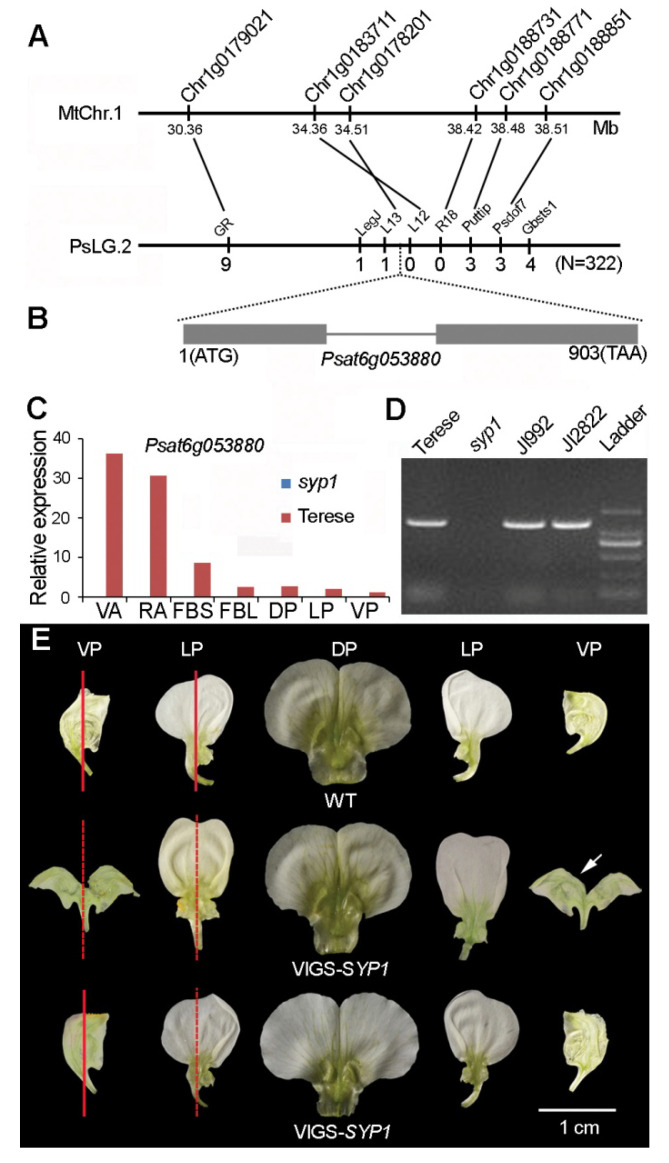
Molecular characterization of *SYP1.* (**A**) Comparative mapping and syntenic analysis of *syp1* in pea and *Medicago truncatula*. The dotted lines indicate the homologous markers in the syntenic region. (**B**) The gene structure of *Psat6g053880*. The black boxes represent the exons and the black line represents the intron. (**C**) The gene expression levels in the different organs of the *syp1* mutant and the wild-type plants. (**D**) PCR amplification of the genomic fragment of *Psat6g053880* in Terese, the *syp1* mutant, JI992, and JI2822. (**E**) The lateral petals and ventral petals of the wild-type plants and VIGS-*SYP1* silenced plants with strong and weak phenotypes. The red lines indicate the IN asymmetry and the dotted lines indicate the abolishment of IN asymmetry. VA, vegetative apices; RA, reproductive apices RA; FBS, 2-mm floral buds; FBL, 5-mm floral buds; DP, the dorsal petal; LP, the lateral petal; VP, the ventral petal.

A comparative genomics approach was carried out to clone the *SYP1* gene. The *syp1* mapping population was developed from a cross between the *syp1* mutant and the JI992 accession. The initial mapping of *syp1* identified linkage with the gene-specific markers LegJ and Puttip on the pea linkage group 2 (chromosome 6; Figure 2A). Furthermore, *SYP1* co-segregated with the newly developed dCAPs marker L13 and R18 in the F_2_ population (Supplementary Table S1). Based on information from the pea marker database (PMD Version 2) and comparative genomics analysis between pea and *Medicago truncatula* [25,26], we found that the *syp1* region shared good synteny with a 4.15 Mb region on chromosome 1 of *M. truncatula* A17 (Figure 2B).

Taking advantage of the good synteny between *M. truncatula* chromosome 1 and pea chromosome 6 [26], we performed quantitative PCR to assess whether there were significant differences in the expression of any of these genes between the wild-type plants and the *syp1* mutants in the syntenic interval [27]. We observed that only one gene in the mapping interval showed significant differences in expression levels between the wild-type plants and the *syp1* mutants in all detected tissues/organs. *Psat6g053880* completely lost its expression in the *syp1* mutant compared to the wild-type plant (Figure 2C). Considering the known function of its tomato and rice homologs in controlling flower and inflorescence development [8,11], we considered *Psat6g053880* as a candidate for *SYP1*. The full-length sequence of the candidate gene in pea was cloned and the sequences of the wild type and the *syp1* mutant were analyzed. We found that this gene was completely deleted in the *syp1* mutant (Figure 2D). In the F_2_ population, 322 plants out of 1387 individuals exhibited the *syp1* mutant phenotype. PCR analysis showed that these 322 plants were homozygous for the deletion of *Psat6g053880* (the primers are listed in Supplementary Table S1), suggesting that *Psat6g053880* gene co-segregated with the *syp1* mutant phenotype.

To further verify that this candidate is the *SYP1* gene, we conducted VIGS assays [23]. The silencing constructs containing different fragments of the gene were infiltrated into the leaves of the wild-type plants (accession JI992). In VIGS-*SYP1* silenced plants, 25.50% and 31.05% of flowers displayed complete or partial defects of IN asymmetry, respectively (Figure 2E). Thus, we concluded that *SYP1* was the candidate gene.

### 2.3. ALOG Family Transcription Factors in Pea

**Figure 3:**
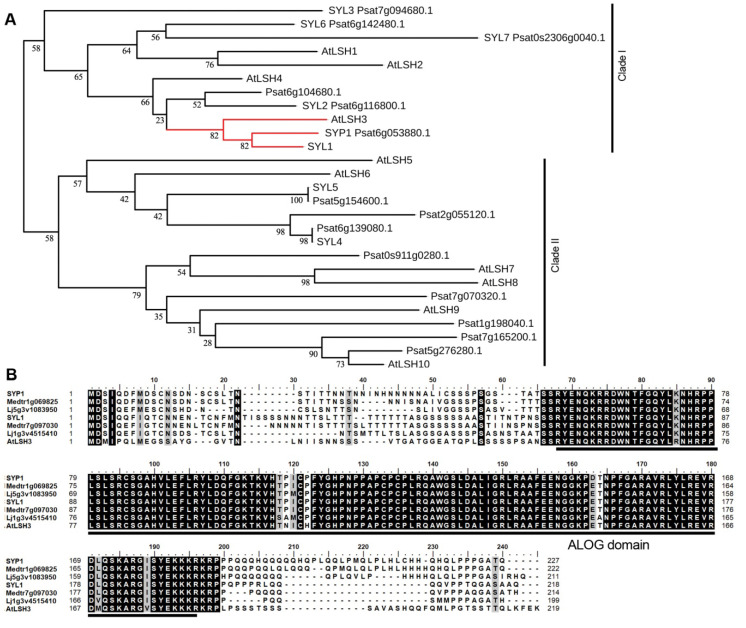
Phylogenetic analysis and sequence alignments of ALOG proteins. (**A**) The neighbor-joining tree of members of the ALOG gene family in pea and Arabidopsis. The bootstrapping value is located in each node as percentages along the branches. Red line indicates the branch including AtLSH3, SYP1 and SYL1. (**B**) The alignment of SYP1, SYL1, Medtr1g069825, Medtr7g097030, Lj5g3v1083950, Lj1g3v4515410, and AtLSH3 proteins using full-length amino acid sequences. The ALOG domain is indicated by a black line.

Sequence alignment showed that *SYP1* contains one intron and two exons and encodes a 227-aa protein (Figure 2B). Phylogenetic analysis indicated that the SYP1 protein belongs to the ALOG family of transcription factors, with high similarity to AtLSH3 in Arabidopsis (Figure 3A). Multiple alignments showed that the encoded protein has a highly conserved ALOG domain at the central part, like other proteins in the ALOG family (also termed DUF640 in Pfam, a comprehensive database of protein domain families, Figure 3B).

To better characterize the ALOG family transcription factors in pea, we conducted a BLASTp search against our local database and a public database [26,27,28]. The searches identified 17 putative ALOG proteins in pea (Figure 3A; Supplementary Table S2). Phylogenetic analysis showed that these ALOG family proteins in pea could be divided into two clades, with SYP1 and SYP1-like (SYL) 1/2/3/6/7/Psat6g104680.1 in clade I and the other ten proteins in clade II (Figure 3A). Clade I and clade II could be further divided into several subclades (Figure 3A). The above results suggested that the ALOG family genes have undergone multiple duplication events during legume evolution.

**Figure 4:**
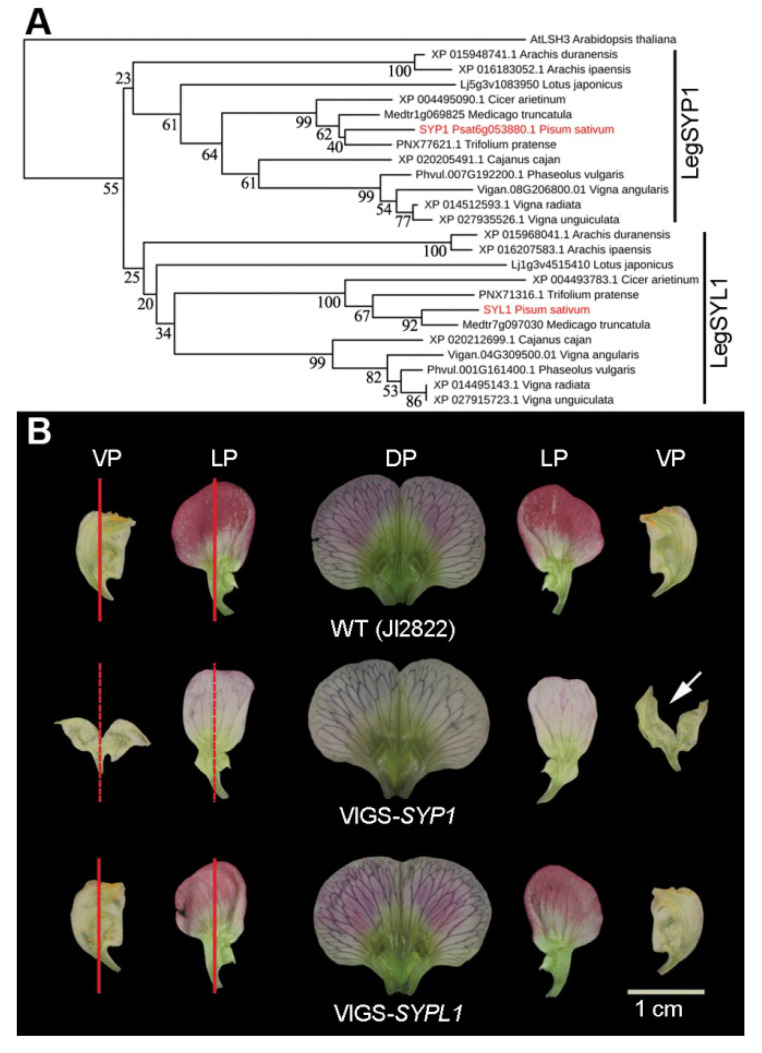
Phylogenetic analysis of LSH3 orthologs in legumes. (**A**) The neighbor-joining tree of AtLSH3 orthologs in legume plants. The bootstrapping value is located in each node as percentages along the branches. Red lines indicate two proteins in pea, SYP1 and SYL1. (**B**) The lateral petals and ventral petals of the wild type and VIGS-*SYP1* and VIGS-*SYL1* silenced plants. The red lines indicate the IN asymmetry and the dotted lines indicate the abolishment of IN asymmetry. The arrow indicates where the ventral petal was cut to flatten the petal. DP, dorsal petal; LP, lateral petal; VP, ventral petal.

To further investigate the possible origin of Arabidopsis LSH3 orthologs in legumes, we identified the orthologs in legume genomes from a number of available databases, including genome-derived and transcriptomic sequences (Supplementary Table S3) and inferred a phylogenetic tree of aligned legume LSH3 orthologs (Figure 4A). Due to duplication of gene block with more than four colinear genes [29], there are two copies of genes encoding the orthologs of LSH3 in legumes, such as pea, *M. truncatula*, mung bean, common bean (*Phaseolus vulgaris*), and wild peanut (*Arachis ipaensis*), forming legume SYP1 (LegSYP1) and LegSYL1 sister branches (Figure 4A).

To test whether the closely related *SYL1* gene is also involved in the control of IN asymmetry, VIGS-*SYL1* silencing constructs were applied to the wild-type plants (accession JI2822). In VIGS-*SYL1* silenced plants, there was no visible alteration of floral organ internal asymmetry in the petals, in contrast to that of the VIGS-*SYP1* silenced plants (Figure 4B), suggesting that *SYL1* is not involved in the control of IN asymmetry.

### 2.4. Expression and Protein Localization of COCH and SYP1

**Figure 5:**
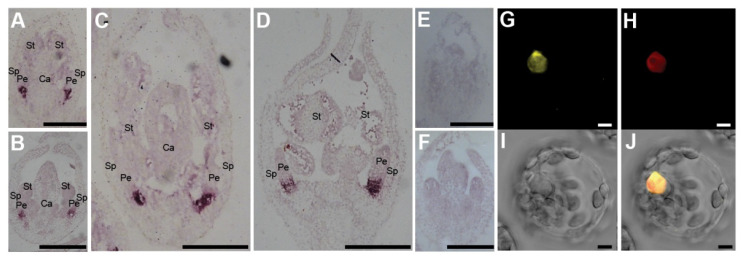
Spatiotemporal expression pattern and subcellular localization of SYP1. (**A**,**C**) *SYP1* gene expression was detected in developing flowers. (**B**,**D**) *COCH* gene expression was detected in developing flowers. (**E,F**) The *SYP1* and *COCH* sense probes were used as the negative controls. No hybridization signal was detected in developing flowers. St, Stamen; Sp, Sepal; Pe, Petal; Ca, Carpel. (**A**–**F**) Scale bars = 100 μm. (**G**–**J**) Subcellular localization of *SYP1* fusion proteins in mung bean protoplasts by YFP or mCherry fluorescence. The fluorescent fusion proteins were expressed in mung bean protoplasts and visualized by confocal microscopy. (**G**–**J**) Subcellular localization analysis of SYP1-YFP together with the nuclear marker OsARF4-mCherry. The cells were analyzed for yellow fluorescence emission, mCherry fluorescence emission, and under bright-field illumination 12 h after transformation. Scale bar = 5 μm.

We explored the expression patterns of *COCH*, *SYP1*, and *SYL1* across different tissues and organs of pea using the data from the gene expression atlas in pea (Supplementary Figure S2A) and our local expression data (Supplementary Figure S2B) [27,28]. *COCH*, *SYP1*, and *SYL1* had similar expression patterns and were expressed in all tissues and organs examined, with the highest level of expression in the nodules, vegetative apices, and reproductive apices (Supplementary Figure S2). To address the molecular function of *SYP1* and COCH in petal development, we performed RNA in situ hybridization analysis. *COCH* and SYP1 genes were expressed in basal regions of the petals throughout petal development (Figure 5A–F). In addition, reverse transcription quantitative PCR (RT-qPCR) analysis of the expression of *SYP1* and *COCH* in the *coch* and *syp1* mutants suggests that *COCH* and *SYP1* do not appear to regulate each other’s transcript levels.

As both SYP1 and COCH may possibly function in transcriptional regulation, we tested whether they might be targeted to the nucleus. The full-length coding regions of *SYP1* and *COCH* were cloned upstream of *YFP* and *CFP*, respectively, in a fusion construct for constitutive expression from the CaMV 35S promoter. Plasmids containing *Pro35S::SYP1-YFP* were transformed into mung bean protoplasts, and the fluorescent protein fusions were then visualized by confocal microscopy. As shown in Figure 5G–J, SYP1-YFP fluorescence was associated with the nucleus, similar to ARF4-mCherry, the positive control [30]. Plasmids containing *Pro35S::COCH-CFP* and *Pro35S::SYP1-YFP* were transiently transformed into *N. benthamiana* leaves, which were then monitored for YFP and CFP fluorescence by confocal microscopy. As shown in Supplementary Figure S3, COCH-YFP fluorescence was associated with the nucleus similar to SYP1-YFP fluorescence.

### 2.5. Physical Interaction between SYP1 and COCH

**Figure 6:**
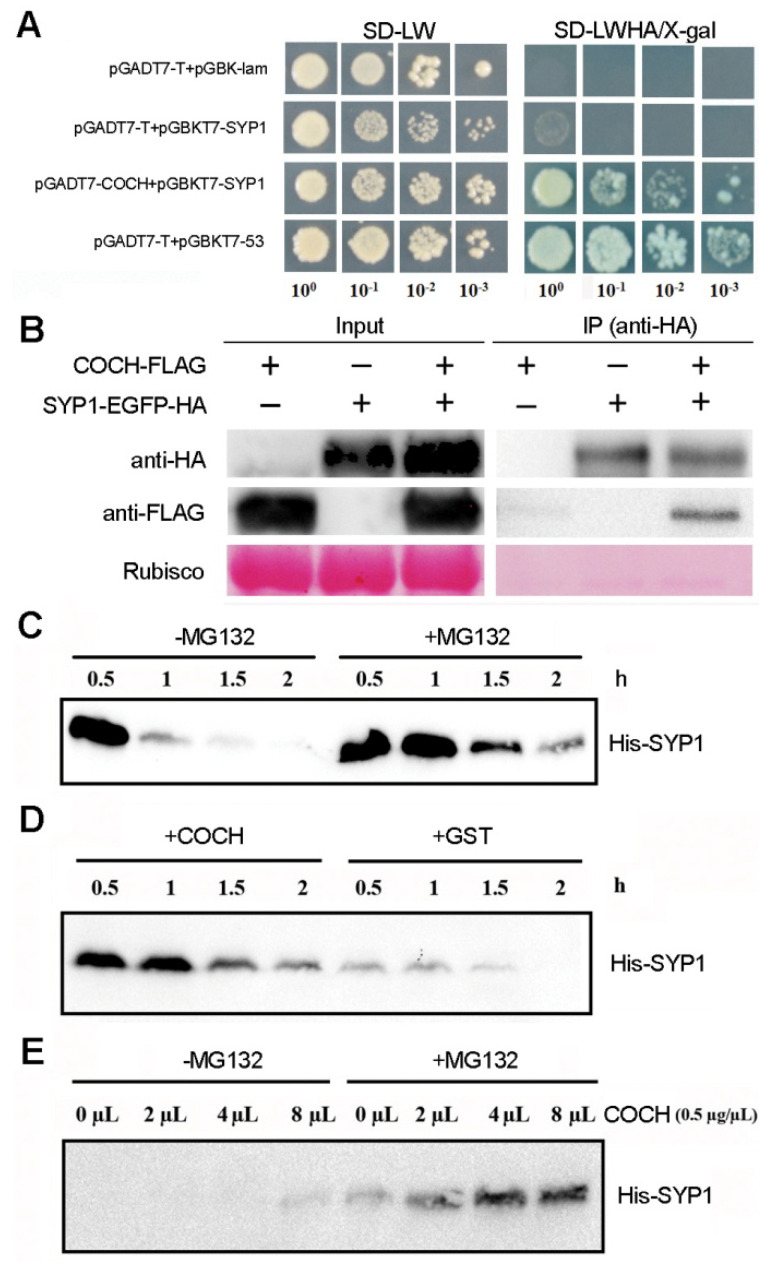
COCH interacted with SYP1. (**A**) Yeast two-hybrid assays for COCH and SYP1. The bait protein was expressed as a binding domain fusion and the indicated prey proteins were expressed as activating domain fusions in yeast AH109 cells. Transformed yeast was grown on selective media lacking Leu and Trp (-2) or lacking Ade, His, Leu, and Trp (-4) plus X-α-Gal to test protein interaction. 10^0^, 10^−1^, 10^−2^ and 10^−3^ represent dilution series. (**B**) COCH was co-immunoprecipitated with SYP1. (**C**) Degradation assay of His-SYP1 performed in a pea cell-free system with or without MG132. (**D**) Degradation assay of His-SYP1 performed in a pea cell-free system with COCH or GST. (**E**) Degradation assay of His-SYP1 performed in a pea cell-free system with or without MG132 and different amounts of COCH.

The ALOG family protein TMF can physically interact with BOP-like proteins in tomato to control organ development [17]. This raised the question of whether COCH might directly interact with SYP1 to regulate the development of petal IN asymmetry and nodulation. Yeast two-hybrid (Y2H) assays showed that there was direct interaction between COCH and SYP1 (Figure 6A). To confirm and demonstrate this interaction in vivo, we conducted co-immunoprecipitation (CoIP) assays. 35S::COCH-FLAG and/or 35S::PsSYP1-EGFP-HA were transiently expressed in Arabidopsis protoplasts, and then crude protein extracts from the cells were immunoprecipitated and analyzed by Western blotting with the anti-FLAG and anti-HA antibodies. As shown in Figure 6B, when both COCH-FLAG and SYP1-HA are co-infected into Arabidopsis protoplast cells, and HA immunoprecipitation is carried out, both HA and FLAG are detected by Western blot, confirming the physical interaction between COCH and SYP1 in vivo.

### 2.6. COCH Represses the Decrease in SYP1 Levels Mediated by the 26S Proteasome

The BOP proteins act as adaptors of CULLIN3 (CUL3)-RING ubiquitin ligases (CRL3) to regulate the abundance of its targets such as PHYTOCHROME INTERACTING FACTOR 4 (PIF4) and LEAFY (LFY) [31,32]. We therefore tested the hypothesis that SYP1 abundance is controlled by COCH in pea. To test this possibility, we first performed a Western blot to examine the protein stability of SYP1 in a cell-free system. His-SYP1 depletion was observed in the absence of the 26S proteasome inhibitor MG132, whereas His-SYP1 was much more stable in the presence of MG132, indicating that the degradation of SYP1 is mediated by the 26S proteasome and degradation of SYP1 could be inhibited by MG132 (Figure 6C). Then we asked whether COCH might regulate SYP1 stability in pea. We found that His-SYP1 was more stable in the presence of the GST-tagged COCH than that with the control GST protein, indicating that COCH might be involved in stabilizing SYP1 (Figure 6D). Then, we determined whether the COCH-mediated regulation of SYP1 protein stability might proceed through the 26S proteasome pathway. His-SYP1 stability increased with increasing amounts of GST-tagged COCH protein, indicating that COCH could attenuate the 26S proteasome-mediated degradation of SYP1 (Figure 6E).

## 3. Discussion

### 3.1. COCH and SYP1 Are Involved in the IN Asymmetry of Floral Organs

It has been reported that COCH is a master regulator of compound leaf and nodule identity [20,21]. In this study, we found that COCH also plays a pivotal role in petal IN asymmetry. The flowers in the *coch* mutants displayed symmetrical lateral and ventral petals similar to the *syp1* mutant (Figure 1). VIGS-*COCH* silenced plants also displayed symmetrical lateral and ventral petals (Supplementary Figure S1). Consistent with this, mutants of the *COCH* ortholog in *L. japonicus* exhibit similar abnormal flowers with symmetric lateral and ventral petals [24], suggesting that the *COCH* orthologs in legumes play conserved roles in petal internal asymmetry. Furthermore, our results indicated that *COCH* genetically interacts with *SYP1* in the control of petal internal asymmetry.

Interestingly, *SYP1* is also involved in regulating nodule development in addition to controlling IN asymmetry. Recently, we found that a root-like structure developed in an apical position on the nodules in the *syp1* mutant, similar to that of the *coch* mutant.

### 3.2. SYP1 Encodes an ALOG Family Protein

We cloned the *SYP1* gene using a comparative genomics approach. Phylogenetic analysis showed that SYP1 is a member of the ALOG family proteins, with high similarity to Arabidopsis LSH3, which is involved in organ boundary development [16]. In rice, the ALOG family gene *G1* regulates the growth of the sterile lemma in the spikelet [8]. In tomato, the ALOG family gene *TMF* controls flower and leaf development [11,17]. Recently, it has been reported that *TfALOG3* in *T. fournieri* controls corolla neck differentiation [19]. The above results suggest that the ALOG family genes play conserved roles in organ development in plants.

Bioinformatics analysis showed that there were a total of 17 putative ALOG proteins in pea and these proteins could be divided into two clades with several subclades (Figure 3). Due to gene duplication [29], there were two copies of genes encoding the orthologs to LSH3 in legumes, forming legume SYP1 (LegSYP1) and LegSYL1 branches (Figure 4). The gene expression atlas in pea showed that *SYP1* and *SYL1* had similar expression patterns and were expressed in all tissues and organs examined (Supplementary Figure S2). However, in VIGS-*SYL1* silenced plants, there was no visible alteration of IN asymmetry in the petals of lateral or ventral petals, in contrast with the VIGS-*SYP1* silenced plants (Figure 4), suggesting functional divergence between *SYP1* and *SYL1* after gene duplication.

### 3.3. COCH Interacts with and Promotes SYP1 Stability

The tomato ALOG protein TMF physically interacts with BOP-like factors to control organ development [17]. Y2H assays and CoIP assays confirmed that COCH and SYP1 proteins physically interact (Figure 6). Recently, the BOP proteins have been shown to act as substrate adaptors in a CRL3 ubiquitin ligase complex to regulate the abundance of its targets, such as PIF4 and LFY [31,32]. As for PIF4, BOP2 promotes its degradation in response to light [31], while BOP2 positively controls LFY protein levels and activity in Arabidopsis [32]. In this study, we discovered that COCH could interact with SYP1 and repress the degradation of SYP1 (Figure 6). Algorithms for the prediction of protein ubiquitination sites (UbPred) [33] identified a putative ubiquitination site in SYP1 (K149) with medium confidence.

Our results suggest that SYP1 undergoes post-translational regulation and a conserved module is recruited to control the development of multiple organs in plants. In the future, identifying CRL3 orthologs in pea and investigating how SYP1 activity is regulated by CRL3 complexes should shed more light on zygomorphic flower development in legumes.

## 4. Materials and Methods

### 4.1. Plant Materials and Growth Conditions

The pea (*P. sativum* L.) *syp1* mutant (JI3515) was identified from a fast neutron mutant library in the accession Terese from Institut J.P. Bourgin, INRA, Versailles, France [3]. The classical *coch* mutant (JI2757) and the wild type (JI116) were obtained from the pea germplasm repository at John Innes Center, Norwich, UK [20,21]. The plants were grown at 20 to 22 °C with a 16:8 h light:dark photoperiod at 200 μmol m^−2^ s^−1^.

### 4.2. Comparative Mapping and Molecular Cloning

Genetic mapping in pea was performed as described previously [5]. We crossed the *syp1* mutants with pollen from the wild-type plants (accession JI992) and all F1 plants showed a wild-type phenotype. F2 mapping population was developed and mutant lines were screened.

For the initial mapping of the *syp1* locus, we used polymorphic markers between *syp1* and JI992 across all linkage groups in pea [34,35]. For fine mapping of *syp1*, we developed new markers in the syntenic region according to sequences of isolated genes in the accessions JI992 and Terese, based on comparative genomic and transcriptomic analyses. Primer sets used for molecular cloning and amplification by PCR are listed in Supplementary Table S1.

### 4.3. RNA Extraction and Reverse Transcription Quantitative PCR (RT-qPCR)

Total RNA was isolated from pea plant tissues and organs as described previously [36]. Reverse transcription was conducted as described in the manufacturer’s manual (TaKaRa Biotechnology, Dalian, China). qPCR was carried out using Power SYBR Green Master mix (Applied Biosystems, Foster City, CA, USA) and the Roche LightCycler 480 (Roche, Basel, Switzerland) according to the manufacturer’s manual. The primers used for qPCR are listed in Supplementary Table S1.

### 4.4. RNA In Situ Hybridization

For in situ probes, the transcripts of *SYP1* and *COCH* were amplified with primers from cDNA fragments (Supplementary Table S1). RNA in situ hybridization was conducted as described [37].

### 4.5. Subcellular Localization Assays

For the subcellular localization assays, the full-length coding sequences of *SYP1* and *COCH* were amplified by PCR (primers are listed in Supplementary Table S1) and subcloned into the pA7-YFP and pA7-CFP vector, respectively. Mung bean (*Vigna radiata*) protoplasts were prepared and DNA-polyethylene glycol (PEG)-calcium transformation was conducted as described previously [6,38]. The transformed protoplasts were visualized by a confocal fluorescence microscope (Leica, TCS SP5, Wetzlar, Germany). For the analysis of the co-localization of SYP1 and COCH, the fluorescent fusion proteins were transiently expressed in the leaves of 6-week-old *Nicotiana benthamiana* plants, as described previously [39], and were visualized by confocal microscopy (Leica, TCS SP5).

### 4.6. Yeast Two-Hybrid (Y2H) Assays

The GAL4 Y2H assays were performed following the manufacturer’s manual (Clontech). The full-length coding sequences of *COCH* and *SYP1* were amplified by PCR (the primers are listed in Supplementary Table S1) and then cloned into the pGBKT7 and pGADT7 vectors, respectively. The plasmids of GAL4-AD and GAL4-BD fused to the related proteins were co-transformed into yeast strain AH109 and the clones were screened on double selective media (SD/-Leu-Trp) and quadruple selective media (SD/-Leu-Trp-Ala-His) with X-α-gal (80 mg/mL).

### 4.7. Co-immunoprecipitation (CoIP) Assays

For the CoIP assays, full-length coding sequences of *SYP1* and *COCH* were amplified by PCR (the primers are listed in Supplementary Table S1) and subcloned into the pHBT-EGFP-HA and pHBT-FLAG vectors, respectively. The *A. thaliana* mesophyll protoplast isolation and transformation were performed as previously described [38]. Six hours after the transformation, the Arabidopsis protoplasts were collected by centrifugation and ground in liquid nitrogen. Protoplasts were treated with lysis buffer [38]. Extracts were centrifuged at 13,000 rpm for 10 min at 4 °C and 50 μL of the supernatant was taken out as the input sample. The remaining supernatant was incubated with anti-HA agarose beads (Sigma A2095, Shanghai, China) for 3 h at 4 °C with gentle rotation. After incubation, beads were collected by centrifugation at 5000 rpm for 30 s at 4 °C, and then washed four times with wash buffer (10 mM HEPES, pH 7.5, 100 mM NaCl, 1 mM EDTA, 10% glycerol, and 1% Triton X-100). Input and immunoprecipitated samples were added to 5× SDS protein loading buffer, mixed well, and heated for 10 min at 95 °C, followed by sodium dodecyl sulfate polyacrylamide gel electrophoresis (SDS-PAGE) and Western blotting with anti-HA antibodies (Sigma A8592) and anti-FLAG antibodies (Sigma H6533). Three independent biological repeats were performed.

### 4.8. Expression of Proteins in Escherichia coli

His(6)-tagged SYP1 and GST-tagged COCH were expressed in *E. coli* BL21 using the pET-28b and pMAL-c2x vectors, respectively, and then the proteins were purified with the kit according to the manufacturer’s protocol (Qiagen, Dusseldorf, Germany).

### 4.9. Protein Extraction and Cell-Free Degradation Assay

Fresh total protein was extracted from two-week-old wild-type pea leaves in protein extraction buffer at 4 °C as described [40]. The degradation assay of His-tagged SYP1 protein was performed in a pea cell-free system. Equal volumes of total protein from pea leaves were incubated with an equal amount of His-SYP1 purified from *E. coli* with or without the 26S proteasome inhibitor MG132. +MG132, final concentration 50 mM; −MG132, equal volume of DMSO as a negative control. Samples were collected at 0.5, 1, 1.5, and 2 h after incubation at 30 °C and then subjected to immunoblot analysis using the anti-His antibody. The degradation assay of His-SYP1 was performed in a pea cell-free system with COCH or GST (negative control) as described above.

### 4.10. Virus-Induced Gene Silencing (VIGS) Assays

The VIGS assays were conducted in pea as described previously [23]. The accessions JI992 and JI2822 from the pea germplasm repository at John Innes Center were used for VIGS assays in this study. The primers used for the VIGS-*COCH*, VIGS-*SYP1*, and VIGS-*SYL1* constructs are listed in Supplementary Table S1.

### 4.11. Phylogenetic Analysis

The amino acid sequences were aligned using MEGA6 [41]. The phylogenetic trees were built using the neighbor-joining method and tested with the bootstrap method with 1000 bootstrap replicates, and the bootstrap values are denoted on the nodes.

## Supplementary Materials

The following are available online at https://www.mdpi.com/1422-0067/21/11/4060/s1, Table S1: Primers Used in this Study, Table S2: ALOG Proteins in Pea and Arabidopsis, Table S3: AtLSH3 Orthologs in Legumes, Figure S1: Phenotype of *COCH* VIGS-silenced Pea Plants. The lateral petals and ventral petals of the wild type, and the strong and weak phenotypes of VIGS-*COCH* silenced plants. The red lines indicate the IN asymmetry and the dotted lines indicate the abolishment of IN asymmetry. The arrow indicates where the ventral petal was cut to flatten the petal. DP, dorsal petal; LP, lateral petal; VP, ventral petal, Figure S2: Expression Patterns of *SYP1* (*PsCam046169*), *SYL1* (*PsCam000959*), and *COCH* (*PsCam0036654*). 01_PsUniLC_RootSys_A_HN, Root system, stage A, High-nitrate; 02_PsUniLC_RootSys_A_LN, Root system, stage A, Low-nitrate; 03_PsUniLC_Root_B_LN, Roots, stage B, Low-nitrate; 04_PsUniLC_Root_F_LN, Roots, stage F, Low-nitrate; 05_PsUniLC_Nodule_A_LN, Nodules, stage A, Low-nitrate; 06_PsUniLC_Nodule_B_LN, Nodules, stage B, Low-nitrate; 07_PsUniLC_Nodule_G_LN, Nodules, stage G, Low-nitrate; 08_PsUniLC_Shoot_A_HN, Shoot, stage A, High-nitrate; 09_PsUniLC_Shoot_A_LN, Shoot, stage A, Low-nitrate; 10_PsUniLC_Leaf_B_LN, Leaves, stage B, Low-nitrate; 11_PsUniLC_LowerLeaf_C_LN, Lower leaves, stage C, Low-nitrate; 12_PsUniLC_UpperLeaf_C_LN, Upper leaves, stage C, Low-nitrate; 13_PsUniLC_Tendril_BC_LN, Tendrils, stage B+C, Low-nitrate; 14_PsUniLC_Stem_BC_LN, Stems, stage B+C, Low-nitrate; 15_PsUniLC_Peduncle_C_LN, Peduncles, stage C, Low-nitrate; 16_PsUniLC_ApicNode_B_LN, Apical node, stage B, Low-nitrate; 17_PsUniLC_Flower_B_LN, Flowers, stage B, Low-nitrate; 18_PsUniLC_Pods_C_LN, Pods, stage C, Low-nitrate; 19_PsUniLC_Seeds_12dap, Seeds, stage E, High-nitrate; 20_PsUniLC_Seed_5dai, Seeds, stage D, Figure S3: Subcellular Localization of SYP1 and COCH Fusion Proteins in *N. benthamiana* Mesophyll Cells by YFP or CFP Fluorescence. The fluorescent fusion proteins were transiently expressed in *N. benthamiana* mesophyll cells and visualized by confocal microscopy. Cells were analyzed for yellow fluorescence emission, CFP fluorescence emission, and DAPI fluorescence emission 72 h after transformation. Scale bar = 5 μm.
