# Supplementary material for: SYMMETRIC PETALS 1 Encodes an ALOG Domain Protein that Controls Floral Organ Internal Asymmetry in Pea (Pisum sativum L.)
Source: Int J Mol Sci. 2020 Jun 5;21(11):4060. doi: 10.3390/ijms21114060 (PMC7313044; doi:10.3390/ijms21114060)
Supplement: Supplementary file 1 [file ijms-21-04060-s001.pdf]

Supplmantary Materials

Table S1. Primers Used in this Study.

Primer Name	Sequence (5′ to 3′)
for analysis of *SYP1* deletion	F: TGGTGACAAAGATGAAGAGGCA
	R: TCCCTCACTCACTCTTGTCACTG
for VIGS	
SYP1	F1: CCATGGTCAATTCAAGATTTTATGGAC
	R1: AGATCTTACTGTCCAAAGGTGTTCCA
SYP1	F2: CCATGGGTACTTGGATCAATTTGGC
	R2: AGATCTATGGATTTGTATCTGGTTTT
SYP1	F3: CCATGGGAAGAAAATGGAGGAAAACC
	R3: AGATCTGTGACAAAGATGAAGAGGCA
COCH	F1: TCTAGATGTAATGAAAGTGCTACTAGCAT
	R1: GAATTCACGTCTCATCCGACGGATC
COCH	F2: TCTAGAAGCTTGCATTGCTCACTCAG
	R2: GAATTCTCTTCGAGGTCTGCTGCAG
SYL1	F: GACTAGTGGCAAGAGGGATTAGTTATGAG
	R: GAAGATCTTTCATCTCAGTGTACAGCAACC
for in situ	
SYP1	F: GATATCCATCAACAGCAACAACAGCAT
	R: GGATCCGCATCAAGCAGAAAACATACATA
COCH	F: TCGAAGGCCGTTTAGTCCAC
	R: AACCCCAAAGTATCTCGCGG
for Y2H	
SYP1	F: CATATGGATTCAATTCAAGATTTTATGG
	R: GGATCCTTATTGAGTTGCACCTGGAGG
COCH	F: GGAATTCATGTCCCTTGAAGACTCCCTAAGATCTCT
	R: CGGGATCCTTAGTAGTCATGAGAATGATGATGATGATACATGGATG
for CoIP	
SYP1	F: CGCggatccATGGATTCAATTCAAGATTTTATG
	R: CGCggatccTTGAGTTGCACCTGGAGGTGGA
COCH	F: CGCggatccATGTCCCTTGAAGACTCCCTAAG
	R: GTAGTCATGAGAATGATGATGATGAT
for protein expression	
SYP1	F: CATATGGATTCAATTCAAGATTTTATGG
	R: GGATCCTTATTGAGTTGCACCTGGAGG
COCH	F: CGCGGATCCATGTCCCTTGAAGAC
	R: CCGCTCGAGTTAGTAGTCATGAGA
for qRT-PCR	
SYP1	F: GAGAACCAGAAACGCCGTGA
	R: GGGTTTGGGTGCCCATAGAA
COCH	F: GAACTCAGTGGGCTACGAGG
	R: AACCCCAAAGTATCTCGCGG
SYL1	F: CACCGTCCTCCACTTTCTCTT
	R: AGACGGCCTATCAGTGCATC
for *syp1* mapping	
R18	F: TTTCTCGCCACCAAATACCT
	R: AACTTCTGGAGTGACGCTGA
Puttip	F: CATGCTTTCTCACTATTTGCCGC
	R: GCAACCAAAGGTTGATGTTGAGG
L12	F: GACCCAGTAGTTGTGAGAAGGA
	R: CAAGTATGACATTGAGCTGCC
L13	F: AGCAGAGCCTCATACTGGTCC
	R: GATATCAGAGAAGTGTGAACTCGA
LegJ	F: ATCGGAGTCTCTTCGCTGTATCCTCTGCA
	R: GTGGGAACCCAGAAACAGAG
GR	F: TAACCATTTCAATTTCCGAAGC
	R: AACTATTTTTATTTGATCAATTATATTGTCT
AA372	F: GAGTGACCAAAGTTTTGTGAA
	R: CCTTGAACCCATTTTTAAGAGT
AB72	F: ATCTCATGTTCAACTTGCAACCTTTA
	R: CTTCAAAACACGCAAGTTTTCTGA

Gbsts1	F: GTCAACTGGCAACCCAATCT
	R: CACTGTAAGTCCACATTATGCC
for subcellular localization	
SYP1	F: ACGCGTCGACATGGATTCAATTCAAGATTT
	R: CGCGGATCCTTGAGTTGCACCTGGAGGTG
COCH	F: ACGCGTCGACATGTCCCTTGAAGAC
	R: TCCCCCGGGTTAGTAGTCATGAGA

Table S2. ALOG Proteins in Pea and Arabidopsis.

**The ALOG family proteins in pea and Arabidopsis**
>Psat6g104680.1<br>MMNSLQEFESSSNNKDMINTNPMSIIVSSSSSSSSMMTISSTTSASSSTATTTSPSSPSTTTPSRYENQKRRDWNTFGQYLRN<br>HRPPLSLSRCSGAHVLEFLRYLDQFGKTKVHTQICPFFGHPNPPAPCPCPLRQAWGSLDALIGRLRAAFEENGGKPEAN<br>PFGARAVRLYLREVRDSQAKARGISYEKKKRKRPPQPPPPPPPSNATGTDQS
>Psat5g276280.1<br>MMNSLQEFESSSNNKDMINTNPMSIIVSSSSSSSSMMTISSTTSASSSTATTTSPSSPSTTTPSRYENQKRRDWNTFGQYLNN<br>LRPPVPISQCNSNHVLEFLRYLDQFGKTKVHLQGCMFYGQPEPPAPCTCPLRQAWGSLDALIGRLRAAYEENGGTPETN<br>PFASGAIRVFLREVRECQAKARGIPYKKKKKSTGNQSKGNDESSSSTMHFS
>Psat7g165200.1<br>MSSSGSNIHGKNIMEQESPTIPTTVTPSRYESQKRRDWNTFGQYLNNMRPPVQLSQCNCNHVLDFLRYLDQFGKTKVHL<br>QGCMFYGQPEPPAPCTCPLRQAWGSLDALIGRLRAAYEENGGSPDTNPFASASIRVYLREIRECQAKARGIPYKKKKKGS<br>QSKGNDESSSTSMNFS
>Psat1g198040.1<br>MSISMQNDTVQASSSSSRPGATADHPSVVAPLSRYESQKKRDWNTFGQYLRNQTPPVSLSQCNFNHVLEFLRYLDQFGK<br>TKVHLHGCIFFGQPDPPAPCTCPLRQAWGSLDALIGRLRAAYEEHGGSPENNPFGTGAIRVYLREVKECQSKARGIPYTK<br>KKKKRSQIKGTHNNSSKSFKQLAS
>Psat0s911g0280.1<br>MSNKGKDIVDGSSSRSSTSTIGDGNGGGDHHQQQQIPLSRYESQKRRDWNTFGQYLRNQRPSVALSQCNSNHVLEFLR<br>YLDQFGKTKVHLQGCLFFGQTEPPGPCTCPLKQAWGSLDALIGRLRAAYEENGGLPETNPFASGSIRIYLREVRDSQAKA<br>RGIPYKKKKKKRIPIKHNGDTSNLPMQ
>Psat7g070320.1<br>MKKLQRVRYESQKRRDWNTFGQYLKSQNPPVPLSNCSFNHVLDFLRYLDQFGKTKVHLNGCIFFGQPTPPAPCACPLK<br>QAWGSLDALIGRLRAAYEENGGSPQTNPFAGGAVRVFLREVKDSQQKARGIPYKKKKKKKKSYQVKGSTSSTAQTQQS<br>AYVNQQDS
>Psat6g139080.1<br>MRILQKVHRPYMKLASGGRTTTCQPSSTPQPELHQRRPTPKADTSHKSDEELEPRSNTKVPRRLGCPYFGQPNPPSPCAC<br>PLKQAWGSLDALIGRLRAAFEENGGKQESNPFGTKAVRIYLREVREGQAKARGIPYEKKKRKGSTVTTAAIVSTAAAVV<br>TGDGNTNRIELGVTPYNSVSLSAEIV
>Psat2g055120.1<br>QVHVTGSYCGQQTPSPCACPLKQAWGSLDALIGRLRAAFEENGGKQESNPFGTKAVRIYLREVREGQAKARGIPYEKK<br>KRKRSTVTTAAIVSTAAAVVTGDGNTNRIELGVTPNNSVSLSAEIV
>SYP1 Psat6g053880<br>MDSIQDFMDSCNSDNSCSLTNSTITTNNTNNINHNNNNNALICSSSPSGSTATSSRYENQKRRDWNTFGQYLKNHRPPL<br>SLSRCSGAHVLEFLRYLDQFGKTKVHTPICPFYGHPNPPAPCPCPLRQAWGSLDALIGRLRAAFEENGGKPDTNPFGAR<br>AVRLYLREVRDLQSKARGISYEKKKRKRPPPQQQHQQQQQHQPLQQLPMQLPLHLCHHQHQLPPPGATQ
>SYL1<br>MDSIQQFIQTCNNENTCNFMNTISSSSNNNNTTSLTTTTTTTTTASGSSSSSAASTITNTPNSSSRYENQKRRDWNTFGQYLK<br>NHRPPLSLSRCSGAHVLEFLRYLDQFGKTKVHTPICPFYGHPNPPAPCPCPLRQAWGSLDALIGRLRAAFEENGGKPET<br>NPFGARAVRLYLREVRDLQSKARGISYEKKKRKRPPQPPPRLQQQQVPPTQGASAAQ
>SYL2 Psat6g116800<br>MNSLEEFNSSNNTQTKSIINFTTIATTSEDKNITNFTSSSSSSSAPQPANTLSRYENQKRRDWNTFGQYLRNHRPPLSLSRC<br>SGAHVLEFLRYLDQFGKTKVHSQICPFFGHPNPPAPCPCPLRQAWGSLDALIGRLRAAFEENGGKPEDNPFGARAVRLF<br>LREVRDSQSKARGISYEKKKRKRPQQQQQLPPPSNNAT
>SYL3 Psat7g094680<br>MSAAVAAAASAAISGCQNSSSSSSNHSEELITSQRMRVSVAPPLSRYESQKRRDWNTFGQYLKNHRPPLTLSRCSGAHVL<br>EFLRYLDQFGKTKVHSENCAYFGNSHPPGPCPCPLKQAWGSLDALIGRLRAAFEENGGSSEMNPFGARAVRLYLREVR<br>DAQAKARGIAYEKKKRKKVNVQNQPNGSMMVDQDHGIVHSGYGYGGGYVDQYSNFNGIASRNDAAVSYFSS
>SYL4<br>MEAASGGEPPPPTPSAQPQPDSSPPTPTPSRYESQKRRDWNTFQQYLPNHKPPLTLARCSGAHVIEFLKYLDQFGKTKVH<br>VTGCPYFGQPNPPSPCACPLKQAWGSLDALIGRLRAAFEENGGKQESNPFGTKAVRIYLREVREGQAKARGIPYEKKKR<br>KRSTVTTAAIVSTAAAVVTGDGNTNRIELGVTPNNSVSLSAEIV

>SYL5
MDSSSGGAVAGASSSEPPTTSPTVTVTTVQPEGSSPPPPPQAPPSRYESQKRRDWNTFLQYLQNHKPPLTLARCSGAHVIE
FLKYLDQFGKTKVHVSGCPYFGHPNPPAPCACPLKQAWGSLDALIGRLRAAYEENGGRPESNPFGAKAVRIYLREVRE
GQAKARGIPYEKKKRKRSAVTVSAVSSSGGGANDDSGGGNGGAGDNTVVGGGTTATTTTAANASTS

>Psat5g154600
MLLHFSGIGAMPWIIMSEIKCNVDDSAVGVPINAACLGIFRDHNSNHLGSFSLNIGQGNALFVEITGAIMAIEITKDNHW
THLCGAHVIEFLKYLDQFGKTKVHVSGCPYFGHPNPPAPCACPLKQAWGSLDALIGRLRAAYEENGGRPESNPFGAKA
VRIYLREVREGQAKARGIPYEKKKRKRSAVTVSAVSSSGGGANDDSGGGNGGAGDNTVVGGGTTATTTTAANASTS

>SYL6 Psat6g142480
MDLVSQSTNSSLSKIESSTSGSSITTTNNSVSSGSSTPTSSRYENQKRRDWNTFCQYLRNHRPPLSLALCSGAHVLEFLNYL
DQFGKTKVHHHPCPFFGIPNPPAPCPCPLRQAWGSLDALIGRLRAAYEENGGRPETNPFGSRAVRIYLRDVRDFQAKAR
GVSYEKKRKRPKPKITAPTTAR

>SYL7 Psat0s2306g0040
MELVSESTNQKGSENGIKSNRYECQKRRDWNTFCQYLRNHRPPLSASMCNGSHVLEFLHYLDQFGKTKVHNPNCPFFG
MPTPPAPCPCPLRQAWGSLDALIGRLRAAYDQAVNGGNHINPFGDGVVRFYLRDVRDFQSKARGVSYHKKRKRPNRN
IVAPP

>AtLSH1
MDLISHQPNKNPNSSTQLTPPSSSRYENQKRRDWNTFCQYLRNHRPPLSLPSCSGAHVLEFLRYLDQFGKTKVHHQNC
AFFGLPNPPAPCPCPLRQAWGSLDALIGRLRAAYEENGGPPEANPFGSRAVRLFLREVRDFQAKARGVSYEKKRKRVNR
QKPQTQPPLQLQQQQQPQQGQSMMANYSGATV

>AtLSH2
MDLISQNHNNRNPNTSLSTQTPSSFSSPPSSSRYENQKRRDWNTFCQYLRNHHPPLSLASCSGAHVLDFLRYLDQFGKT
KVHHQNCAFFGLPNPPAPCPCPLRQAWGSLDALIGRLRAAYEENGGAPETSPFGSRSVRIFLREVRDFQAKSRGVSYEK
KRKRVNNKQITQSQPQSQPPLPQQPQQEQGQSMMANYHHGATQ

>AtLSH3
MDMIPQLMEGSSAYGGVTNLNIISNNSSSVTGATGGEATQPLSSSSSPSANSSRYENQKRRDWNTFGQYLRNHRPPLSLS
RCSGAHVLEFLRYLDQFGKTKVHTNICHFYGHPNPPAPCPCPLRQAWGSLDALIGRLRAAFEENGGKPETNPFGARAV
RLYLREVRDMQSKARGVSYEKKKRKRPLPSSSTSSSSAVASHQQFQMLPGTSSTTQLKFEK

>AtLSH4
MDHIIGFMGTTNMSHNTNLMIAAAATTTTTSSSSSSSSGGSGTNQLSRYENQKRRDWNTFGQYLRNHRPPLSLSRCSGA
HVLEFLRYLDQFGKTKVHTHLCPFFGHPNPPAPCACPLRQAWGSLDALIGRLRAAFEENGGSPETNPFGARAVRLYLRE
VRDSQAKARGISYEKKKRKRPPPPLPPAQPAISSSPN

>AtLSH5
MEGETAAKAAASSSSSPSRYESQKRRDWNTFLQYLRNHKPPLNLSRCSGAHVLEFLKYLDQFGKTKVHATACPFFGQP
NPPSQCTCPLKQAWGSLDALIGRLRAAFEEIGGGLPESNPFAAKAVRIYLKEVRQTQAKARGIPYDKKKRKRPHTDTAT
PIAGDGDDAEGSGGAALVVTAATTV

>AtLSH6
MESADSGRSDPVKGDDPGPSFVSSPPATPSRYESQKRRDWNTFLQYLKNHKPPLALSRCSGAHVIEFLKYLDQFGKTKV
HVAACPYFGHQQPPSPCSCPLKQAWGSLDALIGRLRAAYEENGGRPDSNPFAARAVRIYLREVRESQAKARGIPYEKKK
RKRPPTVTTVRVDVASSRQSDGDPCNVGAPSVAEAVPP

>AtLSH7
MASPSNKGKGIAEGSSQPQSQPQPQPHQPQSPPNPPALSRYESQKRRDWNTFCQYLRNQQPPVHISQCGSNHILDFLQY
LDQFGKTKVHIHGCVFFGQVEPAGQCNCPLKQAWGSLDALIGRLRAAFEENGGLPERNPFAGGGIRVFLREVRDSQAK
ARGVPYKKRKKRKKRNPMKSHDGEDGTTGTSSSSNLAS

>AtLSH8
MTSTNTRNKGKCIVEGPPPTLSRYESQKSRDWNTFCQYLMTKMPPVHVWECESNHILDFLQSRDQFGKTKVHIQGCVFF
GQKEPPGECNCPLKQAWGSLDALIGRLRAAYEENGGLTEKNPFARGGIRIFLREVRGSQAKARGVLYKKKEASCSCWY
GN

>AtLSH9
MSSDRHTPTKDPPDHPSSSSNHHKQPLPPQPQQPLSRYESQKRRDWNTFVQYLKSQNPPLMMSQFDYTHVLSFLRYLD
QFGKTKVHHQACVFFGQPDPPGPCTCPLKQAWGSLDALIGRLRAAYEEHGGGSPDTNPFANGSIRVHLREVRESQAKA
RGIPYRKKKRRKTKNEVVVVKKDVANSSTLNQSFT

>AtLSH10
MSSPRERGKSLMESSGSEPPVTPSRYESQKRRDWNTFGQYLKNQRPPVPMSHCSCNHVLDFLRYLDQFGKTKVHVPGC
MFYGQPEPPAPCTCPLRQAWGSLDALIGRLRAAYEENGGPPETNPFASGAIRVYLREVRECQAKARGIPYKKKKKKKPT
PEMGGGREDSSSSSSSFSFS

Table S3. AtLSH3 Orthologs in Legumes.

**LSH3 Orthologs in Legumes**
>SYP1 Psat6g053880.1 [Pisum sativum]<br>MDSIQDFMDSCNSDNSCSLTNSTITTNNTNNINHNNNNNALICSSSPSGSTATSSRYENQKRRDWNTFGQYLKNHRPP<br>LSLSRCSGAHVLEFLRYLDQFGKTKVHTPICPFYGHPNPPAPCPCPLRQAWGSLDALIGRLRAAFEENGGKPDTNPFGA<br>RAVRLYLREVRDLQSKARGISYEKKKRKRPPPQQQHQQQQQHQPLQQLPMQLPLHLCHHQHQLPPPGATQ
>SYL1 [Pisum sativum]<br>MDSIQQFIQTCNNENTCNFMNTISSSSNNNTTSLTTTTTTTTTASGSSSSSAASTITNTPNSSSRYENQKRRDWNTFGQYL<br>KNHRPPLSLSRCSGAHVLEFLRYLDQFGKTKVHTPICPFYGHPNPPAPCPCPLRQAWGSLDALIGRLRAAFEENGGKPE<br>TNPFGARAVRLYLREVRDLQSKARGISYEKKKRKRPPQPPPRLQQQQVPPTQGASAAQ
>AtLSH3 [Arabidopsis thaliana]<br>MDMIPQLMEGSSAYGGVTNLNIISNNSSSVTGATGGEATQPLSSSSSPSANSSRYENQKRRDWNTFGQYLRNHRPPLSLS<br>RCSGAHVLEFLRYLDQFGKTKVHTNICHFYGHPNPPAPCPCPLRQAWGSLDALIGRLRAAFEENGGKPETNPFGARAV<br>RLYLREVRDMQSKARGVSYEKKKRKRPLPSSSTSSSSAVASHQQFQMLPGTSSTTQLKFEK
>Lj5g3v1083950 [Lotus japonicus]<br>MDSIQEFMESCNSHDNTNCSLSNTTSNSLIVGGSSPSASVTTTSSRYENQKRRDWNTFGQYLKNHRPPLSLSRCSGAHVL<br>EFLRYLDQFGKTKVHTPMCPFYGHPNPPAPCPCPLRQAWGSLDALIGRLRAAFEENGGKPETNPFGARAVRLYLREVR<br>DLQSKARGISYEKKKRKRPHPQQQQQQQQPLQVLPHHHHQLQLPPPGASIRHQ
>Lj1g3v4515410 [Lotus japonicus]<br>MDSIQEFIGTCNSDSTCSLTNNTSMTTLTSLASGSGASSSPSASNSTAATSSRYENQKRRDWNTFGQYLKNHRPPLSLSRC<br>SGAHVLEFLRYLDQFGKTKVHSAMCPFYGHPNPPAPCPCPLRQAWGSLDALIGRLRAAFEENGGKPEANPFGARAVR<br>LYLREVRDVQSKARGISYEKKKRKRPPPQQSMMPPPAGATH
>Phvul.007G192200.1 [Phaseolus vulgaris]<br>MDSIQEFMDSCNSHITTTTTAITTTSNSLVGSSNSPSVSPNTSSRYENQKRRDWNTFGQYLKNHRPPLSLSRCSGAHVLEF<br>LRYLDQFGKTKVHTPICPFYGHPNPPAPCPCPLRQAWGSLDALIGRLRAAYEENGGKPEINPFGARAVRLYLREVRELQ<br>SKARGISYEKKKRKRPPPPPLPQQQQPSSLTLPRHHHHHHHLPPPGATQ
>Phvul.001G161400.1 [Phaseolus vulgaris]<br>MDSNIQDFIDTCNSDNTCNLIANSTTTSLTTSATSSSAGSTSITSSSRYENQKRRDWNTFGQYLKNHRPPLSLSRCSGAHV<br>LEFLRYLDQFGKTKVHTPICPFYGHPNPPAPCPCPLRQAWGSLDALIGRLRAAFEENGGKPEANPFGARAVRLYLREVR<br>DLQSKARGISYEKKKRKRPPPQQQPMPLSLPPPGASATH
>XP_020205491.1 [Cajanus cajan]<br>MDSIQEFMESCHSDITNTATTITTSNNSLVGSSNSPSASSTTTSSRYENQKRRDWNTFGQYLKNHRPPLSLSRCSGAHVLE<br>FLRYLDQFGKTKVHTPICPFYGHPNPPAPCPCPLRQAWGSLDALIGRLRAAFEENGGKPETNPFGARAVRLYLREVREL<br>QSKARGISYEKKKRKRPPPPTPQQQQQQHHHQSLPLPHHHLPPPGATQ
>XP_020212699.1 [Cajanus cajan]<br>MDSNIQDFIDTCNSDNTCNLTNTTSILTTSATSSSPSSTSITNTSSRYENQKRRDWNTFGQYLKNHRPPLSLSRCSGAHVL<br>EFLRYLDQFGKTKVHTPICPFYGHPNPPAPCPCPLRQAWGSLDALIGRLRAAFEENGGKPEANPFGARAVRLYLREVR<br>DLQSKARGISYEKKKRKRPQPQQQPMPLPHHPLPPPGASATH
>Medtr1g069825 [Medicago truncatula]<br>MDSIQDFMDSCNSDNSCSLTNSTITTNSSNNNISNAIVGSSSPSGSTTTSSRYENQKRRDWNTFGQYLKNHRPPLSLSRCS<br>GAHVLEFLRYLDQFGKTKVHTPICPFYGHPNPPAPCPCPLRQAWGSLDALIGRLRAAFEENGGKPETNPFGARAVRLY<br>LREVRDLQSKARGISYEKKKRKRPPQQQPQQLQLQQQQPMQLQLPLHLHHHHQHQLPPPGATQ
>Medtr7g097030 [Medicago truncatula]<br>MDSIQEFIGTCNNENLTCNFMNNNNNNNTISTTTTSLTTTTTTASGSSSSSAASTIINSPNSSSRYENQKRRDWNTFGQYLK<br>NHRPPLSLSRCSGAHVLEFLRYLDQFGKTKVHTPICPFYGHPNPPAPCPCPLRQAWGSLDALIGRLRAAFEENGGKPET<br>NPFGARAVRLYLREVRDLQSKARGISYEKKKRKRPPPQQQQQVPPPAQGASATH
>XP_015948741.1 [Arachis duranensis]<br>MDSSIQEFMESCNSDNNTNNINRNIISNNNNNNSSSIVVGGGGSSPSGGGSSSTTTTTTSTSSSRYENQKRRDWNTFGQYL<br>KNHRPPLSLSRCSGAHVLEFLRYLDQFGKTKVHTPICPFYGHPNPPAPCPCPLRQAWGSLDALIGRLRAAFEENGGKPE<br>ANPFGARAVRLYLREVRDLQSKARGISYEKKKRKRPPQQHNHHQQHQHQHQQQQQTLSMAMPLMPLLNHHHHH<br>HHHHHHQLPPPGATQ
>XP_016183052.1 [Arachis ipaensis]<br>MDSSIQEFMESCNTDNNTNNINRNIISINNNNNNSSSIVVGGGGSSPSGGGSSSTTTTTTSTRSSRYENQKRRDWNTFGQYL<br>KNHRPPLSLSRCSGAHVLEFLRYLDQFGKTKVHTPICPFYGHPNPPAPCPCPLRQAWGSLDALIGRLRAAFEENGGKPE<br>ANPFGARAVRLYLREVRDLQSKARGISYEKKKRKRPPQQHNHHNHHQQHQHQHQQQQQQQQTLSMAMPLMPLLN<br>HHHHHHHHHHHHQLPPPGATQ
>XP_015968041.1 [Arachis duranensis]<br>MDSIQEFMDTCHSAGNTFTTTTTTNNNNNNNTVAGTSGSSSSSPAGSTTSSSRYENQKRRDWNTFGQYLKNHRPPLSLSRC<br>SGAHVLEFLRYLDQFGKTKVHTPICPFYGHPNPPAPCPCPLRQAWGSLDALIGRLRAAFEENGGKPEANPFGARAVRL<br>YLREVRDLQSKARGISYEKKKRKRPPPQQQPQQQQSNIGVGVGVGVGVVLPLHHHMPPPPGARTTHHQ

>XP_016207583.1 [Arachis ipaensis]

MDSIQEFMDTCHSAGNTFTTTTNNNNNTTTVAGTSGSSSSSPAASTTSSSRYENQKRRDWNTFGQYLKNHRPPLSLSRC
SGAHVLEFLRYLDQFGKTKVHTPICPFYGHPNPPAPCPCPLRQAWGSLDALIGRLRAAFEENGGKPEANPFGARAVRL
YLREVRDLQSKARGISYEKKKRKRPPPQQQPQQQPNIGVGVGVVLPLHHHMPPPPGARTTHHQ

>XP_014495143.1 [Vigna radiata]
MDSNIQDFIDTCNSDNTCNLITNTTTTNLTTSATSSSAASTSIASSSRYENQKRRDWNTFGQYLKNHRPPLSLSRCSGAH
VLEFLRYLDQFGKTKVHTPICPFYGHPNPPAPCPCPLRQAWGSLDALIGRLRAAFEENGGKPEANPFGARAVRLYLREV
RDLQSKARGISYEKKKRKRPPPQQQPMPLPHHSLPPPGASATH

>XP_014512593.1 [Vigna radiata]

MDSIQEFMDSCNSHITTTTTAITATTNSLVGTSNSPSASPNTSSRYENQKRRDWNTFGQYLKNHRPPLSLSRCSGAHVLE
FLRYLDQFGKTKVHTPICPFYGHPNPPAPCPCPLRQAWGSLDALIGRLRAAYEENGGKPEINPFGARAVRLYLREVREL
QSKARGISYEKKKRKRPPPPPPPLPQPQQQQSLTLPHHHHHHHHHHLPPSGATQ

>Vigan.04G309500.01 [Vigna angularis]
MDSNIQDFIDTCNSDNTCNLITNTTTTNLTTTASSSSAASNSIASSSRYENQKRRDWNTFGQYLKNHRPPLSLSRCSGAH
VLEFLRYLDQFGKTKVHTPICPFYGHPNPPAPCPCPLRQAWGSLDALIGRLRAAFEENGGKPEANPFGARAVRLYLREV
RDLQSKARGISYEKKKRKRPPPQQQPMPLPHHSLPPPGASATH

>Vigan.08G206800.01 [Vigna angularis]
MDSIQEFMDSCNSHITTTTTAITATTNSLVGTSNSPSASPNTSSRYENQKRRDWNTFGQYLKNHRPPLSLSRCSGAHVLE
FLRYLDQFGKTKVHTPICPFYGHPNPPAPCPCPLRQAWGSLDALIGRLRAAYEENGGKPEINPFGARAVRLYLREVREL
QSKARGISYEKKKRKRPPPPPPPLPQPQQQQSLTLPHHHHHHHHHHLPPSAESFLAVVHVDCCL

>XP_004495090.1 [Cicer arietinum]
MDSLQDFMDSCNSDNSCSLTNSTITTTTNTNSTTTTTNNNTLVCSSSPSASTTTSSRYENQKRRDWNTFGQYLKNHRPP
LSLSRCSGAHVLEFLRYLDQFGKTKVHTPICPFYGHPNPPAPCPCPLRQAWGSLDALIGRLRAAFEENGGKPETNPFGA
RAVRLYLREVRDLQSKARGISYEKKKRKRPPQQQQQQQLQQQHHHHQQQQQQQSMSLPLHLHHHHQHHLPPPG
ATQ

>XP_004493783.1 [Cicer arietinum]
MDSIQEFINTCNIENSCNFMNNNNNSISSTTSLTTTASGSSSSSAVSTITTNTPNSSSRYENQKRRDWNTFGQYLKNHRPPL
SLSRCSGAHVLEFLRYLDQFGKTKVHTPICPFYGHPNPPGPCPCPLRQAWGSLDALIGRLRAAFEENGGKPEANPFGAR
AVRLYLREVRDLQSKARGISYEKKKRKRPPLPTPPPTAANSLDIIN

>PNX77621.1 [Trifolium pratense]
MDSSIQDFMDSCNSDNSCSLTTNSTITTNNNISNSLVGSSSPSGSTTTSSRYENQKRRDWNTFGQYLKNHRPPLSLSRCSG
AHVLEFLRYLDQFGKTKVHTPICPFYGHPNPPAPCPCPLRQAWGSLDALIGRLRAAFEENGGKPDTNPFGARAVRLYL
REVRDLQSKARGISYEKKKRKRPPPQQQQQQQQQHHHQQLPMQLPLHLHHHHQHQLPPPGATQ

>PNX71316.1 [Trifolium pratense]
MDSIQEFIQTCNNENTCNFLNNNSISTTNTTSLTTTATGSSSSSAVSTITNTPNSSSRYENQKRRDWNTFGQYLKNHRPPL
SLSRCSGAHVLEFLRYLDQFGKTKVHTPICPFYGHPNPPAPCPCPLRQAWGSLDALIGRLRAAFEENGGKPETNPFGAR
AVRLYLREVRDLQSKARGISYEKKKRKRPPPQQPLPSSQGASATH

>XP_027935526.1 [Vigna unguiculata]
MDSIQEFMDSCNSHITTTTTAVTATSNSLVGTSNSPSASPNTSSRYENQKRRDWNTFGQYLKNHRPPLSLSRCSGAHVLE
FLRYLDQFGKTKVHTPICPFYGHPNPPAPCPCPLRQAWGSLDALIGRLRAAYEENGGKPEINPFGARAVRLYLREVREL
QSKARGISYEKKKRKRPPPPPPPLPQPPPQQQQSLTLPHHHHHHHHHHHHLPPPGATQ

>XP_027915723.1 [Vigna unguiculata]
MDSNIQDFIDTCNSDNTCNLIANSTTTNLTTSATSSSSAASTSIASSSRYENQKRRDWNTFGQYLKNHRPPLSLSRCSGA
HVLEFLRYLDQFGKTKVHTPICPFYGHPNPPAPCPCPLRQAWGSLDALIGRLRAAFEENGGKPEANPFGARAVRLYLR
EVRDLQSKARGISYEKKKRKRPPPQQQPMPLPHHPLPPPGASATH

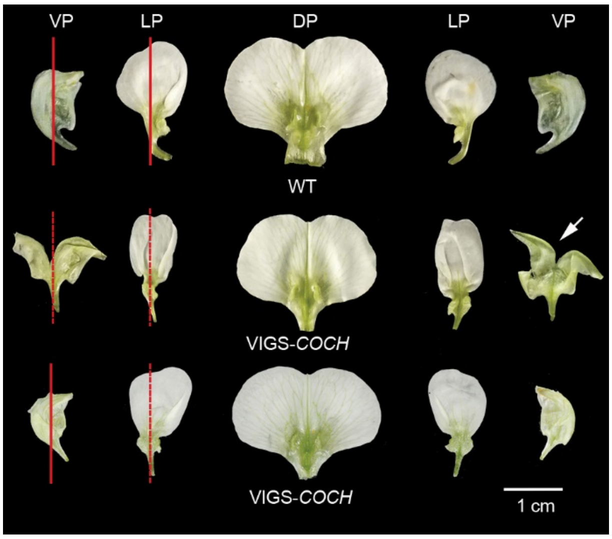

Figure S1. Phenotype of *COCH* VIGS-silenced Pea Plants. The lateral petals and ventral petals of the wild type, and the strong and weak phenotypes of VIGS-*COCH* silenced plants. The red lines indicate the IN asymmetry and the dotted lines indicate the abolishment of IN asymmetry. The arrow indicates where the ventral petal was cut to flatten the petal. DP, dorsal petal; LP, lateral petal; VP, ventral petal.

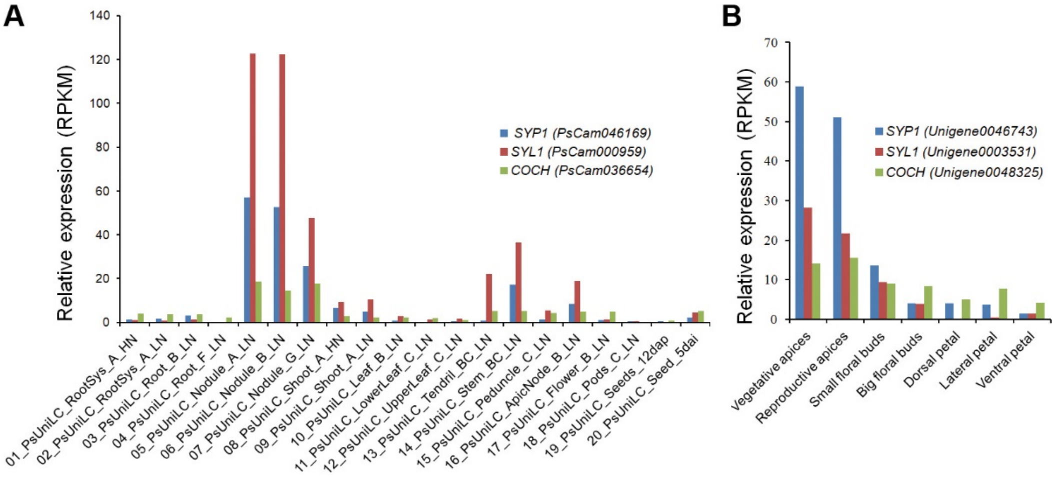

Figure S2. Expression Patterns of *SYP1* (*PsCam046169*), *SYL1* (*PsCam000959*), and *COCH* (*PsCam0036654*). 01_PsUniLC_RootSys_A_HN, Root system, stage A, High-nitrate; 02_PsUniLC_RootSys_A_LN, Root system, stage A, Low-nitrate; 03_PsUniLC_Root_B_LN, Roots, stage B, Low-nitrate; 04_PsUniLC_Root_F_LN, Roots, stage F, Low-nitrate; 05_PsUniLC_Nodule_A_LN, Nodules, stage A, Low-nitrate; 06_PsUniLC_Nodule_B_LN, Nodules, stage B, Low-nitrate; 07_PsUniLC_Nodule_G_LN, Nodules, stage G, Low-nitrate; 08_PsUniLC_Shoot_A_HN, Shoot, stage A, High-nitrate; 09_PsUniLC_Shoot_A_LN, Shoot, stage A, Low-nitrate; 10_PsUniLC_Leaf_B_LN, Leaves, stage B, Low-nitrate; 11_PsUniLC_LowerLeaf_C_LN, Lower leaves, stage C, Low-nitrate; 12_PsUniLC_UpperLeaf_C_LN, Upper leaves, stage C, Low-nitrate; 13_PsUniLC_Tendril_BC_LN, Tendrils, stage B+C, Low-nitrate; 14_PsUniLC_Stem_BC_LN, Stems, stage B+C, Low-nitrate; 15_PsUniLC_Peduncle_C_LN, Peduncles, stage C, Low-nitrate; 16_PsUniLC_ApicNode_B_LN, Apical node, stage B, Low-nitrate; 17_PsUniLC_Flower_B_LN, Flowers, stage B, Low-nitrate; 18_PsUniLC_Pods_C_LN, Pods, stage C, Low-nitrate; 19_PsUniLC_Seeds_12dap, Seeds, stage E, High-nitrate; 20_PsUniLC_Seed_5dai, Seeds, stage D.

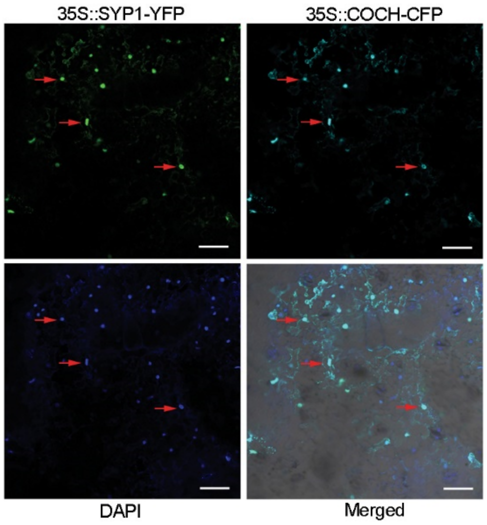

Figure S3. Subcellular Localization of SYP1 and COCH Fusion Proteins in *N. benthamiana* Mesophyll Cells by YFP or CFP Fluorescence. The fluorescent fusion proteins were transiently expressed in *N. benthamiana* mesophyll cells and visualized by confocal microscopy. Cells were analyzed for yellow fluorescence emission, CFP fluorescence emission, and DAPI fluorescence emission 72 h after transformation. Scale bar = 5 μm.
